# New Therapies for Dedifferentiated Papillary Thyroid Cancer

**DOI:** 10.3390/ijms16036153

**Published:** 2015-03-17

**Authors:** Poupak Fallahi, Valeria Mazzi, Roberto Vita, Silvia Martina Ferrari, Gabriele Materazzi, David Galleri, Salvatore Benvenga, Paolo Miccoli, Alessandro Antonelli

**Affiliations:** 1 Department of Clinical and Experimental Medicine, University of Pisa, Via Savi, 10, 56126 Pisa, Italy; E-Mails: poupak@int.med.unipi.it (P.F.); mazzivaleria@gmail.com (V.M.); sm.ferrari@int.med.unipi.it (S.M.F.); 2 Department of Clinical & Experimental Medicine, Section of Endocrinology, University of Messina, Piazza Pugliatti, 1, 98122 Messina, Italy; E-Mails: roberto.vita@yahoo.it (R.V.); sbenvenga@unime.it (S.B.); 3 Department of Surgical, Medical, Molecular Pathology and Critical Area, University of Pisa, Via Savi, 10, 56126 Pisa, Italy; E-Mails: gmaterazzi@yahoo.com (G.M.); galleridavid@hotmail.com (D.G.); paolo.miccoli@dc.unipi.it (P.M.)

**Keywords:** dedifferentiated thyroid cancer, radioiodine, papillary thyroid carcinoma, RET (REarranged during Transfection), *BRAF* (B-Raf proto-oncogene, serine/threonine kinase), *RAS* (Rat Sarcoma), vascular endothelial growth factor receptor 2, vandetanib, cabozantinib, sorafenib

## Abstract

The number of thyroid cancers is increasing. Standard treatment usually includes primary surgery, thyroid-stimulating hormone suppressive therapy, and ablation of the thyroid remnant with radioactive iodine (RAI). Despite the generally good prognosis of thyroid carcinoma, about 5% of patients will develop metastatic disease, which fails to respond to RAI, exhibiting a more aggressive behavior. The lack of specific, effective and well-tolerated drugs, the scarcity of data about the association of multi-targeting drugs, and the limited role of radioiodine for dedifferentiated thyroid cancer, call for further efforts in the field of new drugs development. Rearranged during transfection (RET)/papillary thyroid carcinoma gene rearrangements, *BRAF* (B-RAF proto-oncogene, serine/threonine kinase) gene mutations, *RAS* (rat sarcoma) mutations, and vascular endothelial growth factor receptor 2 angiogenesis pathways are some of the known pathways playing a crucial role in the development of thyroid cancer. Targeted novel compounds have been demonstrated to induce clinical responses and stabilization of disease. Sorafenib has been approved for differentiated thyroid cancer refractory to RAI.

## 1. Introduction

Thyroid cancer is the most common endocrine malignancy, causing approximately 1%–5% of all cancers in females and less than 2% in males [1,2].

New risk factors have emerged in the last decade [3].

Differentiated thyroid carcinomas (DTC), more than 90% of all thyroid tumors, arise from follicular cells, and are classified as papillary (PTC) or follicular (FTC) according to histopathological criteria [2].

In the last decades, an increasing incidence of thyroid cancer (TC) has been reported, in particular for PTC [1]. DTC therapy options are near-total or total thyroidectomy and lymph nodes dissection (in the case lymph nodes are thought to be involved). If the tumor stage of the patients (pts) leads to suppose a significant risk of recurrence or disease-related mortality, subsequent radioiodine ablation is recommended [4]. Thyroid-stimulating hormone (TSH) suppressive therapy is undertaken and annual follow-up based on neck ultrasonography and serum thyroglobulin (Tg) determination are performed [5,6,7].

In pts with no clinically evident residual tumor and with undetectable serum Tg level and negative neck ultrasonography, diagnostic whole-body radioactive iodine (RAI) scan is usually not necessary [4].

DTC show a good prognosis, as more than 85% pts has normal life expectancy [8].

Five percent of pts show distant metastasis at the diagnosis (50% lungs, 25% bones, 20% lungs and bones, 5% other sites). During the follow-up, 10%–15% of pts present recurrent disease (localized in the thyroid bed and lymph nodes), and show a reduction of survival (from 68% to 49% at 10-year); about one third of cancer-related deaths are associated with the presence of neck lesions alone [9].

As the tumor progresses and tumor cells lose the iodide uptake ability, cancer becomes resistant to the traditional therapeutic strategies, and the prognosis worsens significantly [10].

From a histopathological point of view, poorly differentiated thyroid carcinomas (PDTCs) are a subset of thyroid tumors intermediate between DTC and anaplastic thyroid cancers (ATC); Poorly differentiated thyroid carcinomas are more aggressive than DTC, but less than ATC [11,12].

Various molecular changes within PTC cells, such as RET/PTC rearrangements, RAS and BRAF mutations [13], β-catenin mutations [14] underlie the loss of iodide uptake ability.

The aim of this review is to evaluate the state of art of targeted therapies in the approach of dedifferentiated papillary thyroid cancer (DePTC).

## 2. Molecular Pathways Involved in DePTC

### 2.1. RET/PTC Rearrangements, BRAF, RAS, PAX8/PPARγ, Histone Acetylation

**Figure 1:**
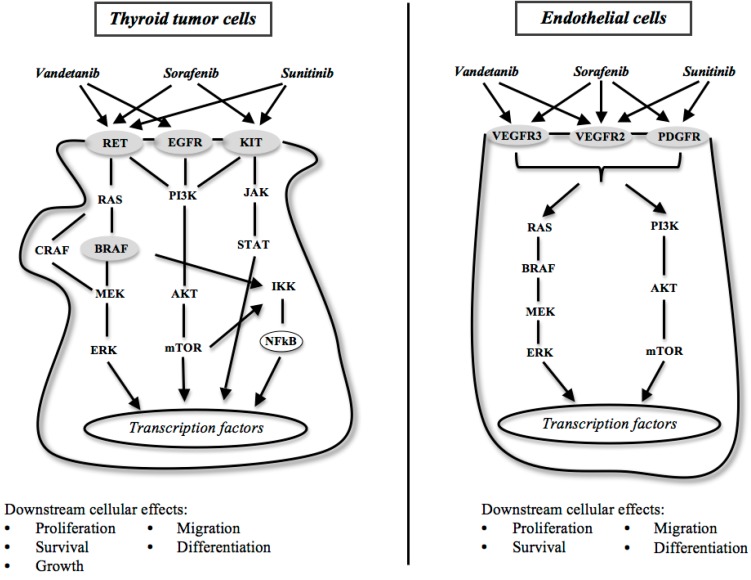
Molecular targets and tyrosine kinase inhibitors in the signaling pathways involved in dedifferentiated papillary thyroid cancer.

RET (REarranged during Transfection), that is involved in cell differentiation, migration and proliferation, is a proto-oncogene located on 10q11.2, and encodes a transmembrane protein whose intracellular region harbors a tyrosine kinase (Figure 1). Activating RET mutations and rearrangements have been found in various human cancer and cancer syndromes [15,16,17].

In particular, an erroneous reparative fusion of the *C*-terminal kinase domain of RET and *N*-terminal domain of an unrelated gene, close to RET, causes RET rearrangements. The resultant chimeric protein dimerizes and autophosphorilates the tyrosine residues of RET. This causes the constitutively activation of the chimeric protein, resulting in an uncontrolled proliferation [18].

For instance, RET/PTC rearrangements are found in up to 40% of adult sporadic PTC [16].

The most frequent rearrangements, RET/PTC (RET gene rearrangements in papillary thyroid carcinomas) (given by the fusion with the *CCDC6* gene, formerly H4) and RET/PTC3 (given by the fusion with the *NCOA4* gene, formerly ELE1) [19] induce thyroid tumors characterized by nuclear grooves and ground glass cells, continuous slow growth rate, and loss of iodide uptake, in transgenic mice similarly to human PTC [20].

Several studies show that thyroid cells exposed to ionizing radiations develops RET/PTC rearrangements, particularly RET/PTC3 [21].

This rearrangement is also associated with the solid variant, a more aggressive phenotype, a greater tumor size, and a more advanced stage at diagnosis, which are all poor prognostic factors [22].

Many authors hypothesize that RET/PTC rearrangements are important for the initiation of the tumor, but are not necessary for its further progression, as RET/PTC rearrangements are frequently found in microcarcinomas, in thyroid adenomas and non neoplastic lesions [23].

BRAF, a member of the RAF family proteins, is a serine-threonine kinase that, upon binding to RAS, phosphorilates MEK (mitogen-activated protein kinase kinase) activating the MAPK (mitogen-activated protein kinases) cascade (Figure 1). Valine to glutamate substitution at residue 600 (V600E) is found in about 45% PTC and rarely in FTC and is correlated with the tumor aggressiveness at presentation, with the risk of tumor recurrence, and with the loss of iodide uptake [18,24].

RAS (“Rat Sarcoma”) is the name given to a gene family, constituted by *K-RAS*, *N-RAS* and *H-RAS*. These genes encode intracellular G-proteins involved in activation of several signaling pathways (Figure 1) [25].

The translocation of the DNA binding domain of PAX8 (Paired box gene 8) to domains A–F of the peroxisome proliferator-activated receptor *(PPAR)γ1* gene is found in 30%–40% of FTC and in 2%–10% of follicular adenomas [26,27]. PAX8/PPARγ rearrangements are less common in the follicular variant of PTC, and rarely are found in the other variants of PTC (0%–1%) [27].

The mechanism of acetylation of NH_2_-terminal lysine residues on histones enhances gene transcription switching chromatin in a more open configuration. When histones are hypoacetylated, chromatin maintains a closed configuration, which hinders gene transcription [28].

### 2.2. Factors Involved in Angiogenesis

By measuring microvascular density, differences in angiogenesis have been related to differences in tumor behavior. Thyroid tumors are more vascular than normal thyroid tissue, and there is a clear correlation between increased angiogenesis and a more aggressive thyroid tumor behavior and metastasis [29].

Experimental evidence has shown that thyroid neoplastic growth and subsequent metastasis formation depend on the tumor’s ability to induce an angiogenic switch, induced by a change in the balance of angiogenic stimulators and inhibitors [30,31].

Some of these are VEGF (vascular endothelial growth factor)/VEGF receptors, EGF (epidermal growth factor)/EGF receptors, PDGF (platelet-derived growth-factor)/PDGF receptors, FGF (fibroblast growth factor)/FGF receptors, HGF (hepatocyte growth factor)/c-Met, and the downstream signaling through Ras-Raf-ERK, Ras-PI3K-AKT-mTOR (Figure 1). Growth factors receptor and the downstream molecules mostly are protein kinases that act by phosphorylation [32].

In the classic model by Hanahan and Weinberg, the molecules are constitutively activated by mutations, rearrangements or amplifications, so that tumor growth is independent from the external growth factors [33].

#### 2.2.1. Vascular Endothelial Growth Factor (VEGF)

VEGF gene family includes VEGF A–C, placental growth factor (PlGF) and PDGF A–D [34]. VEGF is able to mediate endothelial cell adhesion and migration on extracellular matrix, and for this reason is associated with an increased aggressiveness, growth and distant spread of several tumors, including TC [35,36].

VEGF is overexpressed and its main receptor VEGFR-2 is up-regulated in many DTC [28].

In TCs a consistent increase in VEGF, VEGF-C, and angiopoietin-2 was observed. The overexpression of angiopoietin-2 and VEGF was shown in thyroid tumor progression, such as a strong association between tumor size and high levels of VEGF and angiopoietin-2. It has been also shown an increased expression of VEGF-C in lymph node invasive thyroid tumors and, on the other hand, a decrease of thrombospondin-1, an angioinhibitory factor, in thyroid malignancies capable of hematic spread. These results suggest that, in human thyroid tumors, angiogenesis factors are involved in neoplastic growth, progression and aggressiveness [30].

Systemic administration of antiangiogenic drugs that target components of the VEGF-A-VEGF signal transduction pathway (Figure 1) has become a therapeutic option for patients with TC [37].

#### 2.2.2. EGF Receptor (EGFR)

Similarly to VEGF, EGFR (ErbB-1; HER1 in humans) plays a role in TC growth and spread, so that it is highly expressed in aggressive TC (Figure 1). EGFR mutations contribute to RET activation in thyroid cancer [38,39]. In turn, RET/PTC1 and RET/PTC3 up-regulate EGFR expression, with a magnitude of induction similar to that for TSH [38].

The expression of EGFR1 protein is significantly up-regulated in poorly differentiated and ATCs, whereas it is absent or faint in normal thyroid gland tissue and in differentiated thyroid papillary carcinomas, suggesting that up-regulation of EGFR1 expression may be a molecular marker of dedifferentiation in thyroid epithelial carcinomas [40].

High expression of EGFR is associated with lymph node metastasis in PTC, and plays a role in the progression of TC [41,42,43].

More recently, it has been reported a patient with metastatic poorly differentiated thyroid carcinoma with an EGFR mutation who responded to treatment with the selective EGFR TKI (tyrosine kinase inhibitor) erlotinib, strongly suggesting the importance of EGFR as therapeutic target in DePTC [44].

### 2.3. Genomic Analysis

It has been recently described the genomic landscape of 496 PTCs. A low frequency of somatic alterations was observed and it was extended the set of known PTC driver alterations to include *EIF1AX*, *PPM1D*, and *CHEK2* and some gene fusions. The fraction of PTC cases with unknown oncogenic driver was reduced from 25% to 3.5%. Combining the results of the analyses of genomic variants, with gene expression, and methylation it was demonstrated that different driver groups lead to different pathologies with specific differentiation characteristics. It was also proposed a reclassification of TCs into molecular subtypes that better reflect their underlying signaling and differentiation properties. This reclassification could improve the pathological classification of PTC, helping the management of the disease [45].

### 2.4. Tyrosine Kinase Inhibitors (TKIs)

Many strategies have been carried out to block TKI. As RNA interference is difficult to implement, TKI blockade is achieved through monoclonal antibodies against growth factors receptors or TKIs, which act by interfering with the kinase domain-ATP interaction or as allosteric inhibitors [32].

TKIs are emerging as potentially effective options in the treatment of advanced TC. As TKIs are not specific for one kind of tyrosine kinase, we refer to them as multikinase inhibitors. They mostly act on the abovementioned pathways, which, in turn, are involved in angiogenesis, growth, invasiveness, avoidance of apoptosis, and both local and distant spread [46].

Given the fact that TKIs act on pathways that are not selective for a specific malignancy, they have been tested on several tumors, including DTC, medullary thyroid cancer (MTC) and ATC [47].

Individual patient responses are assessed by the response evaluation criteria in solid tumors (RECIST) in the clinical trials evaluating TKIs [48].

Since stable disease in the absence of active treatment is not uncommon, an essential component of the RECIST is that the selection criteria for clinical trials have to include patients who demonstrate measurable disease progression about within 6 months before enrollment. RECIST requires that enrolled patients have measurable target lesions > 2 cm in the largest diameter using conventional imaging techniques or > 1 cm using spiral computed tomography scan, up to a maximum of 5 lesions per organ and 10 lesions in total. Then, the response is analyzed using this baseline evaluation, but it precludes the enrollment of some thyroid cancer patients who do not meet such stringent criteria but may still benefit from targeted therapy [49].

Therefore, a “rapidly progressive disease” has been defined as one showing a >30% tumor growth progression within 12 months.

## 3. Sorafenib

Sorafenib (BAY 43-9006) is a multikinase inhibitor, with a potent activity against RAF, VEGF receptors (VEGFR-2, VEGFR-3), PDGF receptor (PDGFR), c-KIT and RET kinases [50,51].

In preclinical studies, sorafenib has been shown a broad-spectrum antitumor activity in several cancer xenograft models, as colon, breast and non-small-cell lung cancer [50]. Carlomagno *et al.* [52] published a paper on inhibition of oncogenic RET mutants by sorafenib in 2006, showing as the TKI prevented the growth of the TPC1 and TT cell lines, TC cell lines that contain the RET/PTC1 and C634W RET mutation, respectively. This drug, therefore, potentially may inhibit TC growth both through anti-proliferative and anti-angiogenic mechanisms [53].

Sorafenib, actually approved by Food and Drug Administration (FDA) for hepatocellular and renal cell carcinoma, is orally administered at a maximum dose of 400 mg twice daily, and generally is well tolerated. The most frequently reported drug-related adverse events (AE) at any grade included fatigue, anorexia, diarrhea, rash/desquamation and hand–foot syndrome [54]. Given the encouraging results from *in vitro* and *in vivo* trials and the necessity to develop new therapies for iodine-refractory metastatic TC, new studies have been initiated (Table 1).

A phase II trial [55], evaluated the efficacy of sorafenib in 30 pts with metastatic, iodine-nonavid, TC, including differentiated, poorly differentiated, medullary, and anaplastic subtypes. The drug was administrated at 400 mg bid/die; the median duration of treatment was 27 weeks. Six pts (20%) discontinued treatment as a result of AE. Doses were reduced in 47% of pts (14 pts) to control toxicities. A partial response (PR) rate was observed in 23.3% and a stable disease (SD) rate in 53.3%, obtaining a clinical benefit rate (PR plus SD) of 77%, with a median progression-free survival (PFS) of 21 months in pts with DTC. The rate of AE is consistent with other sorafenib trials [55]. 

A few months later, a study was published of 58 pts with metastatic TC, divided in two parts [56]. In the first part, 25 pts were enrolled with metastatic PTC and chemotherapy naive; in the second, patients with PTC, but previously treated with chemoterapy, and other subtype (follicular, Hurthle cell, anaplastic, or mixed thyroid carcinoma) were enrolled. Activating mutations in exon 15 of BRAF was found in 17 of 22 pts with PTC examined. Sorafenib was administrated at 800 mg daily, in two doses, generally well tolerated. This trial showed that sorafenib has clinical and biologic antitumor activity in metastatic PTC, obtaining a PR in 6 pts, while 23 pts had SD longer than 6 months. However, the role of BRAF has not been elucidated definitively [56]. An open-label phase II study of sorafenib at maximum dose (800 mg daily) in metastatic iodine-refractory TC was conducted by Brose *et al.*, in 55 pts with different histological subtypes (47% PTC, 36% FTC/Hürthle Cell, 8% MTC, 9% poorly differentiated/anaplastic) [57]. Genotyping of BRAF was complete in 16 pts. The preliminary results presented at 2009 ASCO annual meeting evidenced an increased PFS in pts with PTC/FTC with B-RafV600E compared to wild-type B-Raf (84 *vs.* 54 weeks, *p* = 0.028). Also, heterogeneity of expression of p-ERK and p-AKT was demonstrated in different tissue areas on treatment [57].

The ability of sorafenib to reinduce RAI uptake in pts with progressive metastatic or locally advanced RAI refractory DTC was evaluated in a prospective phase II study [58]. During 26 weeks, 31 pts received sorafenib 400 mg twice daily. It was observed 59% of clinical beneficial response, 25% of PR and 34% of SD, the estimated PFS was 58 weeks. However, 22% of pts had progressive disease (PD) and diagnostic body scan did not reveal any reinduction of RAI uptake. Sorafenib results were clearly less effective in pts with bone metastases [58].

A paper published in 2010 reports the M. D. Anderson Cancer Center’s experience with the off-label use of the TKIs sorafenib and sunitinib for refractory to iodine DTC (papillary and follicular thyroid cancer) [59]. Sorafenib was used in 13 pts, at the same dose of previous trials described. The results obtained are comparable to other phase II studies evaluating sorafenib in TC (remission rate of 20%, durable response rate of 66%, and a clinical benefit rate of 80%). A longer PFS (19 months) and the median overall survival at 2 years was 67%. In this study, a response variability of the different metastases in the same patient to same therapy was found, with best response in lung and minimal in nonirradiated bone lesions, suggesting a differential expressions and inhibitions of various receptors. Moreover, it has also been evidenced a reduction of Tg levels preceding tumor shrinkage and a correlation between the log Tg and the response to treatment after the start of therapy, suggesting that Tg would be a reliable biologic marker of response to treatment. These data should be validated in larger studies [59].

In another phase II study, sorafenib was administered at 400 mg twice daily in 15 pts with metastatic MTC and 19 pts with locally advanced RAI refractory DTC [60]. The radiological response rate (RR) was 18% for pts with DTC, while the PFS at 2 years was 62% and overall survival 72%. However, 79% of pts required dose reduction for AE (hand–foot syndrome, other skin toxicities, diarrhoea and alopecia). A mutation in BRAF exon 15 was detected in one patient who had demonstrated a dramatic response after 3 months of therapy. Anyway, the sequencing was performed on 10 DTC pts and results were obtained on only 3 of these [60].

In the 2011 ASCO annual meeting the results of UPCC 03305 phase II trial of sorafenib for advanced thyroid carcinoma was presented. Fifty-five pts (85% DTC/PDTCs, 9% ATC, 6% MTC) received the drug at same dose provided in the preceding studies. A longer PFS was observed in pts with DTC/PDTCs (96 *vs.* 93.6 weeks of other thyroid cancer), 38% achieved a PR, 47% had SD. In 66% tissue from patient with DTC/PDTCs was found at least 1 mutation (45% BRAF, 19% RAS, 11% RET, 9% PIK3CA), while in 17% multiple mutation (60% in ATC) [61].

In 2012, data from a retrospective, longitudinal study on use of sorafenib in pts with progressive RAI-refractory DTC was published [62]. Sorafenib has been used off-label in 17 pts, independently from their performance status, at conventional dose. Although the drug was tolerated, 5 fatal events was reported (3 severe bleeding and 2 cardiac arrest), all pts needed dose reductions and/or transient drug interruption to control AE. Thirty percent of pts achieved PR, 41% SD and 18% PD, median PFS was 9 months and median overall survival 10 months. These results probably depended on the general pts conditions at the beginning of the trial, which were worse than other studies. The RR was greater in lymph nodes than lung metastasis, baseline fluorodeoxyglucose-positron emission tomography (FDG-PET) assessment and early FDG-PET response were correlated to radiological response. The FDG-PET could be helpful for the timely identification of non-responding pts, in fact an early reduction in average standardized uptake value was recorded in all pts, but was greater in responding subjects [62].

To evaluate the efficacy of sorafenib in pts with advanced RAI refractory DTC, another phase II trial was conducted on 31 pts receiving sorafenib at 800 mg (400 mg twice daily); the median follow-up and period of treatment was 25 and 9 months, respectively [63]. BRAF V600E was the most observed mutation among the ones evidenced, but not related to disease progression. PR was obtained in 31% and 42% achieved SD after a median follow-up of 25 months. The dose of TKI used was generally well tolerated, although dose reductions were required in 58% of pts, most frequent AE was dermatological [63].

**Table 1:** Clinical trials of Sorafenib in patients with thyroid cancer.

Drug	Thyroid Cancer	Responses				Authors
		PR	SD	PD	PFS (months)	
Sorafenib	30 DeTC	23.3%	53.3%	7%	21	Gupta-Abramson *et al.* [55]
Sorafenib	41 DeTC	15%	56%	–	15	Kloos *et al.* [56]
Sorafenib	31 DeTC	25%	34%	22%	14.5	Hoftijzer *et al.* [58]
Sorafenib	13 DeTC	20%	60%	20%	19	Cabanillas *et al.* [59]
Sorafenib	19 DeTC 15 MTC	18% DeTC 25% MTC	–	–	–	Ahmed *et al.* [60]
Sorafenib	47 DeTC 5 ATC 3 MTC	38% DeTC	47% DeTC	–	23.4	Keefe *et al.* [61]
Sorafenib	17 DeTC	30%	41%	18%	9	Marotta *et al.* [62]
Sorafenib	31 DeTC	31%	42%	–	18	Schneider *et al.* [63]
Sorafenib	207 DeTC	–	–	–	10.8	Brose *et al.* [64]
Sorafenib	8 DeTC	12.5%	62.5%	25%	14–24	Pitoia [65]
Sorafenib Sunitinib Vandetanib	32 DeTC 13 ATC 17 MTC	15% *vs.* 8% DeTC 36% MTC	–	–	6.7 *vs.* 7 DeTC	Massicotte *et al.* [66]

Anaplastic thyroid cancer (ATC); dedifferentiated thyroid cancer (DeTC); medullary thyroid cancer (MTC); partial response (PR); progressive disease (PD); progression-free survival (PFS); stable disease (SD).

A randomized, double-blind, placebo-controlled, phase III trial (DECISION), investigated sorafenib (400 mg orally twice daily) in pts with RAI-refractory locally advanced or metastatic DTC that had progressed [64]. The intention-to-treat population included 417 pts (207 in the sorafenib group and 210 in the placebo group) and the safety population was 416 pts (209 pts instead of 210 in the placebo group). Median PFS was higher in the sorafenib group (10.8 months) than in the placebo group (5.8 months) and improved in all clinical and genetic biomarker subgroups, irrespective of mutation status. AE occurred in 98.6% pts receiving sorafenib: the most frequent were hand-foot skin reaction (76.3%), diarrhoea (68.6%), alopecia (67.1%), and rash or desquamation (50.2%). These results suggest that sorafenib is an effective treatment for pts with RAI-refractory DTC [64].

Also, other more recently published studies suggest a possible role for sorafenib in the treatment of progressive metastatic DTC [65,66].

## 4. Sunitinib

Sunitinib (SU011248) is a small molecule, multitargeted TKI, acting as a selective inhibitor of VEGFR-1, 2, and 3, PDGFR, cKIT, and RET/PTC subtypes 1 and 3, that are involved in signal transduction and growth and their inhibition is determinant in the development of solid tumors [67,68]. Sunitinib is orally administered and is approved for the therapy of clear-cell renal carcinoma and gastrointestinal stromal tumor (GIST) on an intermittent treatment schedule [69], but is under investigation also in other human malignancies. The most common drug-related AE described are fatigue, diarrhea, palmar-plantar erythrodysesthesia, neutropenia, hypothyroidism and hypertension [70].

The antitumoral properties of sunitinib have been investigated by various preclinical studies (Table 2) and Kim *et al.* showed that is a potent inhibitor of RET/PTC oncoproteins *in vitro* [71]. Sunitinib decreases RET/PTC autophosphorylation and STAT3 (signal transducer and activator of transcription) activation, and blocks the transforming capacity of RET/PTC. Furthermore, it exerted a powerful growth-inhibitory effect on the TPC1 cell line, that spontaneously harbors an RET/PTC rearrangement [71].

A study has been performed to investigate the effects of sunitinib in RET/PTC1 rearrangement cells, focusing on signal transduction pathways and gene expression of iodide metabolizing proteins. An increase in sodium-iodide symporter (NIS) gene expression has been demonstrated through the inhibition of MEK/ERK and SAPK/JNK cytoplasmic pathways, individually and in combination, suggesting that blocking these pathways is the mechanism by which sunitinib exerts its direct antiproliferative effect [72].

In another preclinical study the authors investigated the different mechanism of inhibitory effects of sunitinib against RET/PTC rearrangement and BRAF mutation in cell lines and orthotopic TC mouse model [73]. Sunitinib inhibited RET/PTC but not BRAF mutated cells, suggesting that clinical application of sunitinib should be directed by genotyping [73].

The preliminary results of different phase II studies were presented in the ASCO annual meeting in 2008 [74]. In the first trial, 43 pts with evidence of progression of disease (37 DTC, 6 MTC) received sunitinib 50 mg daily on a 4-week-on/2-week-off schedule. Thirty-one pts with DTC completed 2 cycles for evaluation: PR was 13%, SD 68%. For MTC the best response was SD, at 83% [74].

In the second trial, Goulart *et al.* [75] enrolled 18 subjects with metastatic, RAI-refractory and evidence of FDG-PET avid TC (3 MTC, 15 DTC). Sunitinib was administrated at 37.5 mg daily and FDG-PET was performed in 16 pts at baseline and after 7 days of treatment. The FDG-PET response rate was observed in 7 pts, all of them with DTC histology; the RECIST response rate was still being evaluated at the time of report. One patient died for gastrointestinal bleeding [75].

The results for first period of the THYSU study were presented [76]. Sunitinib was given at 50 mg daily for 4 weeks every 6 weeks. Fifteen/17 pts were enrolled (1 ATC, 4 MTC, 8 PTC and 4 other TC) and evaluable for response: 1 pt had a PR, 12 had SD, with 1 pt with >90% decrease of Tg and 1 pt with a dramatic decrease of symptoms [76].

The data from the largest open-label phase II trial was published in 2010 [77], conducted on 28 pts with progressive DTC and 7 with MTC, who received 37.5 mg of sunitinib on continuous basis. A complete response (CR) was observed in 3%, PR in 28% and SD in 46% of subjects. The most common toxicities observed included fatigue (11%), neutropenia (34%), hand/foot syndrome (17%), diarrhea (17%), and leukopenia (31%). Reduction in fluorodeoxyglucose uptake in positron emission tomography was a predictor of PR or stabilization of the disease [77].

Also other more recently published studies suggest a possible role for sunitinib in the treatment of progressive metastatic DTC [66,78].

**Table 2:** Clinical trials of Sunitinib in patients with thyroid cancer.

Drug	Thyroid Cancer	Responses				Authors
		PR	SD	PD	PFS (months)	
Sunitinib	37 DeTC 6 MTC	13% DeTC	68% DeTC 83% MTC	10% DeTC 17% MTC		Cohen *et al.* [74]
Sunitinib	12 DeTC 1 ATC 4 MTC	6%	71%			Ravaud *et al.* [76]
Sunitinib	7 MTC 28 DeTC	28% PR + 3% CR	46%	17%	12.8	Carr *et al.* [77]
Sunitinib	11 DeTC	18% PR + 9% CR	45%	27%	11.5	Dìez *et al.* [78]

Anaplastic thyroid cancer (ATC); complete response (CR); dedifferentiated thyroid cancer (DeTC); medullary thyroid cancer (MTC); partial response (PR); progressive disease (PD); progression-free survival (PFS); stable disease (SD).

## 5. Imatinib

Imatinib (STI571) is a TKI affecting several protein-tyrosine kinases: Bcr-Abl, PDGFR α e β, c-Kit and RET [79], approved by the USA FDA and European Medicines Agency (EMA) for the treatment of chronic myelogenous leukemia, and gastrointestinal stromal tumor. Imatinib has been used in several studies to evaluate the inhibiton of MTC, but no objective response was observed [79,80,81].

In a recent phase I study of imatinib, dacarbazine, and capecitabine in advanced endocrine cancers, no responses were seen in patients with MTC, but 4 of 5 patients experienced SD [82].

## 6. Vandetanib

Vandetanib (ZD6474) (Table 3), is an orally active low-molecular-weight receptor TKI, potent inhibitor of VEGFR-2, targeting also VEGFR-3, EGFR, and RET kinases. The inhibition of RET oncoproteins kinase activity by ZD6474 in RET-mutant cell lines is known since 2002 [83].

Two single-arm phase II clinical trials were developed to evaluate the clinical utility of vandetanib in hereditary MTC, and reported similar preliminary results (20% of pts had a PR while an addictional 53% of pts experienced a SD at 24 weeks [84]; PR was achieved in 16%, SD >24 weeks in 53% [85]).

The ZETA trial [86], an international randomized phase III trial has been performed, comparing ZD6474 (vandetanib 300 mg daily) and placebo in 331 MTC pts: PFS prolongation with vandetanib *vs.* placebo was observed (hazard ratio [HR], 0.46; 95% CI, 0.31 to 0.69; *p* < 0.001).

Vandetanib was approved by FDA as the first TKI to treat adult pts with metastatic or progressive MTC in April 2011 [86].

In a double-blind phase II study [87], 145 pts with locally advanced or metastatic DTC (PTC, FTC, or PDTCS) improved PFS; 72 of them received vandetanib 300 mg/daily and 73 placebo. PFS was higher in pts treated with TKI than with placebo (11.1 and 5.9 months, respectively), PR and SD were 8% and 57%, while for the other group they were 5% and 42%, respectively. The safety and tolerability were consistent with previous studies on vandetanib [87].

Also a more recently published study shows that vandetanib is effective in the treatment of progressive metastatic MTC [66].

**Table 3:** Clinical trials of Vandetanib in patients with thyroid cancer.

Drug	Thyroid Cancer	Responses				Authors
		PR	SD	PD	PFS (months)	
Vandetanib	30 MTC	20%	53%	3%	27.9	Wells *et al.* [84]
Vandetanib	19 MTC	16%	53%	16%	168 days	Robinson *et al.* [85]
Vandetanib	231 MTC	45%	42%	–	–	Wells *et al.* [86]
Vandetanib	145 DeTC	8%	57%	–	11.1	Lebolleux *et al.* [87]

Dedifferentiated thyroid cancer (DeTC); medullary thyroid cancer (MTC); partial response (PR); progressive disease (PD); progression-free survival (PFS); stable disease (SD).

## 7. Motesanib Diphosphate

Motesanib diphosphate (AMG 706) is an ATP-competitive inhibitor of VEGFR1, 2, 3, PDGFR, and Kit. It inhibits human endothelial cell proliferation VEGF-induced, and increases endothelial apoptosis, *in vitro* [88].

The first clinical trial, a phase I study with motesanib diphosphate 125 mg/day orally was conducted in 5 pts affected by DTC, 3 of them had a PR (*i.e.*, >30% reduction in tumor diameters) [89].

Three phase II trials have been carried out in pts with advanced or metastatic, RAI-resistant TC [90,91,92], two of which in pts with MTC [90,92].

In all the three studies, motesanib diphosphate was administered orally 125 mg/day. This daily dose was confirmed to be the best dose to achieve the longest PFS [93].

Sherman *et al.* [90] tested motesanib diphosphate on 93 DTC pts (57 pts, 61% were PTC). A PR was achieved in 14% of them, while SD in 35% for at least 24 weeks. Serum Tg decreased in 81% of pts compared to baseline. The tumor progressed in 7 pts (8%). The median PFS was 40 weeks. The most frequent AE were gastrointestinal (diarrhea, 59%), followed by hypertension (56%), asthenia (46%) and weight loss (40%). Primary hypothyroidism occurred in less than a third of pts (22%). The most common grade 3 side effect was hypertension (25%) [90].

To predict the effectiveness of TKIs, various biomarkers have been identified. Bass *et al.* [92] treated 184 pts (93 DTC and 91 MTC) with motesanib 125 mg/day orally for up to 48 weeks in a phase II trial; 48% of MTC pts achieved SD for at least 24 weeks. Median PFS was 40 weeks for DTC pts, and 48 weeks for MTC pts [92]. Serum PlGF, antagonizing VEGF, increased, while soluble VEGFR2 decreased over the treatment. Both reverted toward baseline at the end of the study. Serum PlGF, soluble VEGFR2 and serum caspase-3/7 activity correlated with the tumor response to motesanib, while baseline serum VEGF correlated with a better prognosis [92].

## 8. Axitinib

Axitinib (AG-013736) is a second-generation inhibitor of VEGFR1, 2, 3, PDGFR and c-Kit [34,94].

Compared to other VEGF-TKIs, it has a greater receptor specificity, particularly against VEGFR2, and for this reason it is the most potent available VEGFR2-TKI [95,96]. It also inhibits endothelial nitric oxide, protein kinase B, ERK, and induces endothelial cells apoptosis that cannot be rescued by exogenous VEGF [97].

Axitinib was more than 10-fold less potent in inhibiting PDGFR and Kit in cell-based assays, compared to the other VEGF-TKIs [98].

The maximum tolerated dose of axitinib assessed in the first phase I study was 5 mg twice daily [99].

In a phase II trial [100], 60 pts with advanced TC, 45 with DTC (30 PTC and 15 FTC) and 11 with MTC, were enrolled and administered with axitinib 5 mg twice daily. SD for at least 16 weeks was achieved in 39% of pts (12 PTC, 7 FTC, 3 MTC), while PR in 29% of pts (8 PTC, 6 FTC, 2 MTC). The median PFS was 18.1 months (72.4 weeks). A negligible effect of axitinib on KIT was confirmed, as a 32%, 35%, 13% decrease of soluble VEGFR2, 3 and soluble Kit, respectively, was evidenced, while serum VEGF was 2.8-fold higher [100].

The most frequent toxicities were fatigue (50%), diarrhea (48%), nausea (33%), anorexia (30%) and a drug-responsive hypertension (28%); the last one was the most common grade 3 side effect (12%). Three pts had grade 4 toxicity, stroke, hypertension, and reversible posterior leukoencephalopathy, respectively. Eight pts (13%) withdrew axitinib because of AE [100].

Furthermore, axitinib did not show a cumulative dose-limiting toxicity [101].

To evaluate the efficacy of TKIs, various biomarkers have been proposed as surrogates, as increase in blood pressure [102], or in erythropoietin blood levels [103].

In a second phase II trial [104], the efficacy and safety of axitinib were evaluated in 52 pts with metastatic or unresectable, locally advanced MTC or DTC, who received a starting dose of axitinib 5 mg twice daily. The overall objective response rate was 35% (18 PR), and 18 pts had SD for ≥16 weeks. The median PFS was 16.1 months, and the median overall survival was 27.2 months. Quality of life was maintained during treatment with axitinib. This study suggests that axitinib could be an additional treatment option for pts with advanced TC [104].

## 9. Cabozantinib

Cabozantinib (XL184), an orally multiple-receptor kinase inhibitor, inhibits VEGFR1, 2, C-MET, RET, c-Kit, FLT3, and Tie-2 [105].

Kurzrock *et al.* conducted a phase I trial on cabozantinib in 37 pts with MTC [106,107]: ten (29%) of 35 MTC pts with measurable disease had a confirmed PR.

Cabanillas *et al.* administered cabozantinib in 15 DTC pts with 140 mg free base (equivalent to 175 mg salt form) daily: 8/15 (53%) had a PR, while 6/15 (40%) had a SD [108].

Considering the abovementioned results and the data obtained also in other studies, FDA has recently approved cabozantinib for the treatment of MTC [109].

## 10. Gefitinib

Gefitinib (ZD1839) is an EGFR-inhibitor firstly used in non-small-cell lung cancer [110], that effectively inhibits ATC proliferation, and induces apoptosis *in vitro* [111].

Pennel *et al.* [112] enrolled 27 pts in a phase II trial, 18 of whom with advanced and RAI-resistant DTC, treated with gefitinib 250 mg/day orally. The most frequent toxicities were cutaneous (rash in 52% of pts) and gastro-intestinal (diarrhea in 41%, anorexia 11%, nausea 9%). The most frequent side effects were rash (7%) and diarrhea (4%). Although no patient achieved PR, 48% of pts attained SD at 3 months, while 24% and 12% at 6 and 12 months, respectively. Five/15 pts (33%) with measurable serum Tg, had a remarkable decrease of Tg levels (<90%) for >6 months [112].

The inactivation of EGFR enhances the cytotoxic effect of doxorubicin and decreases its extrusion; for this reason, the association of gefitinib and doxorubicin has been suggested for the treatment of metastatic FTC, and ATC [113].

Recently, a case-report on a 79-year-old male with metastatic PDTC, with an EGFR mutation, who responded to treatment with the selective EGFR TKI erlotinib, has been reported; a PFS of more than 11 months has been shown [44].

## 11. Pazopanib

As motesanib, pazopanib (GW786034) is a VEGFR1, 2, 3, PDGFR and c-Kit inhibitor, approved for the treatment of renal cell carcinoma [114], but, contrary to sumatinib, it does not induce apoptosis in human renal cell carcinoma cell lines *in vitro* [115].

Pazopanib has been evaluated in 39 pts with advanced DTC in a recent phase II trial, of whom 37 were assessed. At the dose of 800 mg/day orally, a PR was obtained in 18 pts (49%), though no CR was reported; 22 pts (59%) had a PD. There were no differences between PTC and FTC. Tg decreased by at least 30% in 28/32 pts (88%) [116].

The most common toxicities were fatigue (57%), cutaneous (skin/hair hypopigmentation 59%, alopecia 35%), nausea (51%), diarrhea (43%), vomiting (41%), altered taste (54%), anemia (35%), leucopenia (30%). The most frequent grade 3 toxicities were raised ALT levels (11%), lower gastrointestinal hemorrhage (8%). In 23 pts (62%) TSH serum levels raised by more than two times. The median PFS was 11.7 months [116].

More recently it was reported that combining pazopanib with microtubule inhibitors (paclitaxel) produced synergistic antitumor effects in ATC cells and xenografts that were associated with potentiated mitotic catastrophe. Pazopanib potently inhibited aurora A, with pazopanib/paclitaxel synergy recapitulated by aurora A short hairpin RNA knockdown or by specific aurora A pharmacological inhibition. Pazopanib/paclitaxel synergy was reversed by aurora A knockdown. A durable benefit resulted from pilot clinical translation of pazopanib/paclitaxel therapy in a patient with metastatic ATC. These results suggested that the pazopanib/paclitaxel combination is promising in the therapeutic approach in ATC [117].

## 12. Lenvatinib

Levantinib (E7080) is an orally active multi-targeted TKI, which acts on VEGFR1, 2, 3, PDGFR β, RET, c-KIT and FGFR1, 2, 3, 4. Lenvatinib does not significantly inhibit tumor cell proliferation, but it exerts its action on migration and invasion [118].

E7080 has been demonstrated to suppress lymph node and lung metastases in a mammary tumor model [119].

Sherman *et al.* administered lenvatinib in 58 pts with advanced DTC 24 mg/day orally in a phase II trial [120]. Twenty-nine pts had a PR, while median PFS was 12.6 months. The most common AE were hypertension (64%), fatigue (55%) and diarrhea (45%) [120].

## 13. BRAF Inhibitors

BRAF mutations are associated with lymph node metastases, extrathyroidal extension, tumor size, and multifocality in PTC [121]. BRAF activation, through TGF-β and the inhibition of Pax8, leads to the inhibition of NIS expression [122].

Dabrafenib (GSK2118436) is a potent BRAF kinase inhibitor [123], able to block *in vitro* the growth of BRAFV600E positive melanoma and colon cancer human tumor xenografts [124].

Falchook *et al.* [123] enrolled 184 pts with incurable solid tumors with Val600Glu BRAF mutation (Table 4), 14 of them with PTC, in a phase I trial. They took 300 mg/day of dabrafenib orally. The most common AE were fatigue, pyrexia, skin lesions ranging from hyperkeratosis (26%) or actinic keratosis (10%) through kertao acanthoma or squamous-cell carcinoma (11%—low grade, well-differentiated). Nine/14 pts could be assessed. Three/9 achieved PR (two confirmed) [123].

Vemurafenib (PLX4032), an oral analogue of PLX 4720, inhibits BRAF and is already approved for treatment of advanced melanoma. An ongoing phase II trial is evaluating safety and efficacy of vemurafenib in advanced PTC [125].

Other BRAF inhibitors (CEP-32496) have shown *in vitro* a selective action against BRAF and are expected to be effective and favorable also in TC [126].

Other drugs have been developed to interfere with the downstream RAS/RAF pathway. Most of them act on the mitogen-activated protein kinase kinase (MEK or MAPK/ERK kinase), whose substrates are ERK1/2. *In vitro*, MEK inhibitors inhibit growth of human tumors in mouse xenografts [127].

Selumetinib (AZD6244, ARRY-142886) is an oral MEK1 and MEK2 potent inhibitor tested on 57 pts with advanced solid cancers, two of which with TC. The most common toxicities were rash (74%), diarrhea (56%), nausea (44%). The 50% of maximum tolerated dose (100 mg bid) was well tolerated. A patient with MTC achieved SD for 19 cycles [127].

In another paper [128], 39 pts with iodine-refractory PTC were administered with 100 mg bid of selumetinib in a phase II trial. One patient (3%) experienced partial disease, while 21 (54%) achieved SD. Median PFS was 32 weeks (BRAF V600E > BRAF wild-type). The most frequent AE were rash, fatigue, diarrhea and peripheral edema [128].

Another MEK inhibitor, PD0325901 reduces the growth of PTC cells *in vitro* and in xenograft murine models [129].

**Table 4:** Clinical trials with BRAF inhibitors in patients with thyroid cancer.

Drug	Thyroid Cancer	Responses				Authors
		PR	SD	PD	PFS (months)	
Dabrafenib	14 DeTC	21%	–	–	–	Falchook *et al.* [123]
Selumetinib	2 DeTC	–	100% 19 cycles	–	–	Adjei *et al.* [127]
Selumetinib	39 DeTC	3%	54%	28%	8	Hayes *et al.* [128]

Dedifferentiated thyroid cancer (DeTC); partial response (PR); progressive disease (PD); progression-free survival (PFS); stable disease (SD).

## 14. mTOR Inhibitors

The mammalian target of rapamycin, mTOR, constituted by mTORC1 and mTORC2, is the main downstream effector of the PI3K/Akt pathway. It is a serine/threonine kinase that, through the phosphorylation of a number of proteins (p70S6 kinase, 4EBP1) regulates protein synthesis, metabolism, cell growth and survival [130,131]. PI3K/Akt pathway is involved in the thyroid carcinogenesis [132]. Indeed, the initial factor eIF4E, that binds 4EBP1, is overexpressed in PTC as well as MTC cells, and its levels correlate with the aggressiveness of these tumors [132].

The mTOR inhibitor, rapamycin, decreases TC cell growth and viability *in vitro* [132], and the activation of PI3K/Akt pathway causes the inhibition of iodide uptake by NIS. As expected, PI3K/Akt inhibitors increase iodide uptake.

Everolimus (RAD001) is an orally active rapalog (rapamycin analog) that inhibits mTORC1, upon binding FKBP12 [133,134]. It has been approved by FDA for the treatment of pts with advanced renal carcinoma and tested *in vivo* on MTC [135].

Fury *et al.* [130] administered everolimus plus cisplatin in 30 pts with advanced solid tumors in a recent phase I study. Seven/30 pts had TC (5 DTC, 2 MTC). One patient with PTC completed 14 cycles and achieved SD. The most common grade 3 AE were lymphopenia (36%), fatigue (11%) and hyperglycemia (11%) [130].

The combination of another mTOR inhibitor (Temsirolimus) plus a novel MEK inhibitor (RDEA119) has shown a synergistic effect *in vitro* [136].

## 15. Histone Deacetylase Inhibitors

Vorinostat (suberoylanilide hydroxamic acid) is an oral histone deacetylase inhibitor, already approved by the USA FDA for the treatment of cutaneous T-cell lymphoma [137]. Vorinostat is able to arrest TC cell growth and induce apoptosis *in vitro* [138]. Woyach *et al.* tested vorinostat (starting at 200 mg b.i.d. (twice daily)) in 19 pts with TC (16 DTC, 3 MTC), but no patient had a response [139].

Romidepsin (Depsipeptide) is a bicyclic peptide isolated from Chromobacterium violaceum. Depsipeptide was the first histone deacetylase inhibitor reported to be effective in pts with cutaneous T-cell lymphoma, peripheral T-cell lymphoma, and renal cell carcinoma [140]. It is approved by the USA FDA for the treatment of cutaneous T-cell lymphomas [141]. It increases the expression of Tg and NIS *in vitro* [142].

However, a phase II trial on romidepsin in pts with DTC was closed after the first 20 pts because of the lack of response and a grade 5 sudden death and a grade 4 pulmonary embolus [143].

## 16. Limits and Drug Resistance

TKIs are generally less toxic than cytotoxic chemotherapy, but actually they cause significant side effects, as fatigue, hypertension, cutaneous rash, mucositis, hand-and-foot syndrome, nausea, diarrhea, vomiting. Also thyroid dysfunction is a well-known AE of TKI [144]; severe side effects can require the suspension of the therapy with TKIs.

The efficacy of TKIs in pts with DTC has given contrasting evidence in the clinical trials, probably due to the drug resistance, that could arise from the activation of alternate mitogenic signals [145]. TKIs arrest tumor growth but do not remove tumor cells, acting as antiangiogenetic drugs [34].

Hence, the combination of TKIs has been recently proposed [145], though possible interaction between those are yet to be elucidated [146].

The effectiveness of the treatment could be ameliorated by the possibility to test the sensitivity of primary TC cells from each subject to different TKIs [147,148].

By human tumor cells, disease orientated *in vitro* drug screening has some predictive value for the activity of clinical responses [149,150], and could be useful to prevent the administration of inactive chemotherapeutics to pts [151].

*In* *vitro* chemosensitivity tests permit to predict *in vivo* effectiveness in 60% of cases [152], while a negative chemosensitivity test *in vitro* is associated with a 90% of ineffectiveness of the chemotherapy *in vivo* [150], avoiding the administration of inactive drugs to these pts.

Till nowadays, primary TC cell cultures have been obtained from surgical biopsies performed for therapeutic or diagnostic procedures. Recently, our studies demonstrated that fine-needle aspiration (FNA) cytology overcomes this problem, thanks to the possibility to obtain primary cell culture from FNA samples of ATC (FNA–ANA), and opens the way to the use of FNA–ANA to test the sensitivity to different drugs in each patient, avoiding unnecessary surgical procedures and the administration of inactive therapeutics [147,152,153,154,155,156,157,158,159,160,161,162].

## 17. Alternative Therapeutic Strategies

Other alternative strategies are focused on NIS, that is a plasma membrane glycoprotein sited in the basolateral membrane of thyrocytes, that couples sodium and iodide inward transport in favor of the electrochemical gradient. Iodide is therefore translocated towards the follicular lumen through the apical membrane by pendrine, a chloride-iodide transporter [163]. In thyroid cells, TSH stimulates NIS synthesis, while thiocyanate, perchlorate, estrogens, TNF-α, TNF-β, IFN-γ, IL-1α, IL-1β, IL-6, and TGF-β1 inhibit it [164].

As the expression of NIS is impaired but is still maintained, it is possible to obtain thyroid remnant ablation and the detection of TC relapse or metastasis by RAI. Up to 30% of persistent or metastatic TC loose a number of markers of thyroid cell differentiation [163], as the iodide uptake ability of follicular cells. Even though NIS expression can be quantitatively decreased in TC cells, the loss of the ability to uptake iodide seems the result of a functional impairment of NIS. It has been demonstrated by immunohistochemical studies that NIS may be overexpressed in some cancers [165]. Indeed, recent data showed that: (1) NIS localization is mostly intracellular in some TC cells [165]; (2) various NIS repressor have been identified (NIS-repressor, pituitary tumor transforming gene binding factor) [166,167]; (3) the loss of NIS expression could be explained by pre-transcriptional events (as methylation of the NIS promoter) [168]; (4) NIS may abnormally undergo several post-transcriptional and post-translational modifications (for instance, glycosylation), which are able to inactive it [169]; blockers of methyltransferase alone [170] or in combination [171,172] are able to enhance NIS expression. 

Retinoic acids (RA) are active metabolites of vitamin A able to regulate growth and differentiation of many cell types. RA bind to specific nuclear receptors, the retinoic acid receptors (RAR) and the retinoid X receptors (RXR). Recent studies have shown RA induce *in vitro* re-differentiation of TC cells, increasing expression of NIS, and cellular (131)I uptake. RA also induce anti-proliferative effects, and apoptosis in TC cells. Clinical studies have demonstrated that iodide uptake may be induced after RA in about 20%–50% of pts with DePTC, and long-term follow-up of DePTC pts showed that RA can induce partial tumor regression or at least tumor growth stabilisation [173,174].

PPARγ agonists are another exciting field for redifferentiating therapy of DePTC [10,175,176].

In a clinical study 20 pts with DTC were enrolled in an open-label, phase II trial of oral rosiglitazone treatment. Five of 20 pts had a positive radioiodine scan after rosiglitazone treatment. By RECIST criteria, no patient had a complete or partial response to rosiglitazone treatment at 3 months follow-up. These findings suggest that rosiglitazone therapy may induce radioiodine uptake in some pts with DTC but this did not result in clinically significant response on long-term follow-up [177].

Recently, targeted NIS gene transfer, by viral and nonviral vectors, followed by radionuclide ((131)I, (188)Re, (211)At) therapy, has been recently suggested for the treatment of advanced or DeTC. This intriguing approach has prompted great interest due to the specificity as well as the low toxicity [178].

## 18. Conclusions

Much progress has been made recently in the genetic and molecular studies of DePTC, and the set of known PTC driver alterations was extended (to include *EIF1AX*, *PPM1D*, and *CHEK2* and some gene fusions). A reclassification of TCs into molecular subtypes was also proposed, that could improve the pathological classification of PTC, helping the management of the disease. TKIs are emerging as potentially effective options in the treatment of advanced TC. Sorafenib seems to be a promising therapeutic option in patients with advanced DePTC that are not responsive to traditional therapies. However, the efficacy of TKIs in pts with DTC has given contrasting evidence in the clinical trials, probably due to the drug resistance. Furthermore, TKIs might cause significant side effects (as fatigue, hypertension, cutaneous rash, mucositis, hand-and-foot syndrome, nausea, diarrhea, vomiting, thyroid dysfunctions), and severe side effects can require suspension. Several studies are currently under way to evaluate the long-term efficacy and tolerability of TKIs in DePTC, since progression of TC can be slow. The effectiveness of the treatment could be ameliorated by the possibility to test the sensitivity of primary DePTC cells from each subject to different TKIs. In fact, disease orientated *in vitro* drug screening permit to predict *in vivo* effectiveness in 60% of cases, while a negative chemosensitivity test *in vitro* is associated with a 90% of ineffectiveness of the chemotherapy *in vivo*, avoiding the administration of inactive (potentially toxic) drugs to these pts. Further research is needed to determine the ideal targeted therapy, based on molecular characterization of the tumor and of the host factors, to obtain the best response in terms of survival and quality of life.
